# Brain Distribution and Sexually Dimorphic Expression of Amylin in Different Reproductive Stages of the Zebra Finch (*Taeniopygia guttata*) Suggest Roles of the Neuropeptide in Song Learning and Social Behaviour

**DOI:** 10.3389/fnins.2019.01401

**Published:** 2020-01-13

**Authors:** Gergely Zachar, Catherine Montagnese, Emese A. Fazekas, Róbert G. Kemecsei, Szilvia M. Papp, Fanni Dóra, Éva Renner, András Csillag, Ákos Pogány, Arpád Dobolyi

**Affiliations:** ^1^Department of Anatomy, Histology and Embryology, Semmelweis University, Budapest, Hungary; ^2^MTA-ELTE Laboratory of Molecular and Systems Neurobiology, Department of Physiology and Neurobiology, Eötvös Loránd University and the Hungarian Academy of Sciences, Budapest, Hungary; ^3^Department of Ethology, Eötvös Loránd University, Budapest, Hungary; ^4^Human Brain Tissue Bank and Microdissection Laboratory, Semmelweis University, Budapest, Hungary

**Keywords:** parenting, offspring, song system, avian brain, songbird, vocalisation, hypothalamus, social brain network

## Abstract

The expression of the recently identified neuropeptide, amylin, is restricted in rodents to the postpartum preoptic area and may play a role in the control of parental behaviours and food intake. These processes are substantially different between bird and rodent parents as birds do not lactate but often show biparental care of the offspring. To establish the presence and role of amylin in the bird brain, in the present study, we investigated the distribution of amylin in brains of adult male and female zebra finches in three different reproductive stages (i.e. paired without young, incubating eggs or provisioning nestlings) and in unpaired control birds living in same sex flocks. Amylin mRNA was identified in the hypothalamus of zebra finch by RT-PCR, which was also used to produce probes for *in situ* hybridisation. Subsequently, *in situ* hybridisation histochemistry was performed in brain sections, and the labelling signal was quantified and compared between the groups. Amylin showed a much wider brain distribution than that of rodents. A strong and, in some regions, sexually dimorphic label was found in the striatum and several brain regions of the social behavioural network in both males and females. Many regions responsible for the learning of birdsong also contained amylin-positive neurons, and some regions showed sex differences reflecting the fact that vocalisation is sexually dimorphic in the zebra finch: only males sing. Area X (Ar.X), a striatal song centre present only in males, was labelled in paired but not unpaired male. Ar.X, another song centre, the lateral part of the magnocellular nucleus of the anterior nidopallium (lMAN) also contained amylin and had higher amylin label in paired, as opposed to unpaired birds. The wider distribution of amylin in birds as compared to rodents suggests a more general role of amylin in social or other behaviours in avian species than in mammals. Alternatively, parental care in birds may be a more complex behavioural trait involving a wider set of brain regions. The sex differences in song centres, and the changes with reproductive status suggest a participation of amylin in social behaviours and related changes in the singing of males.

## Introduction

Amylin, or islet amyloid polypeptide, is a 37 amino acid peptide belonging to the calcitonin family of peptides (Hay et al., 2015). It is released from the pancreas and plays a role as a satiating hormone (Leffert et al., 1989; Ogawa et al., 1990). Its peripheral application reduces food intake (Lutz, 2010; Roth, 2013) via the penetration of amylin through the circumventricular organs (Barth et al., 2004; Potes and Lutz, 2010). In the rodent brain, amylin receptors have a relatively widespread distribution (Christopoulos et al., 1995), and have been suggested to play a role in food intake control (Levin and Lutz, 2017), ingestive behaviour (Mietlicki-Baase et al., 2017) and anti-psychotic actions (Baisley et al., 2014). Yet, in rodents, the expression of amylin in the central nervous system has only been found in the preoptic area during a specific reproductive stage – the postpartum period – when a 25-fold increase was detected in mRNA levels compared to non-maternal females (Dobolyi, 2009). The preoptic area is involved in the control of maternal responsiveness as lesion or inhibition of its neurons eliminates maternal behaviours (Dulac et al., 2014). A number of neurons are activated in the preoptic area in rat and mouse dams in response to pup exposure (Numan, 2007; Matsushita et al., 2015). In mother rats, amylin neurons were also shown to be activated by pup exposure suggesting a role of amylin in controlling maternal behaviours (Szabó et al., 2012). In mammals, maternal behaviours co-occur with lactation, which includes profound hormonal changes (Bridges, 2015). Amylin was also induced in maternally sensitised nulliparous rats, which showed maternal behaviour without lactation, but not in mother rats at late pregnant stage implying that amylin is more likely to play a role in the control of maternal behaviours rather than maternal hormonal alterations (Szabó et al., 2012).

There are substantial differences in parental care between birds and mammals. In altricial bird species, parental care can be divided into two phases, incubation and offspring provisioning. In contrast to mammals, parents share the burden of raising the young in most bird species, including the zebra finch (Cockburn, 2006; Remes et al., 2015). While social brain networks have been suggested to be well conserved during evolution (O’Connell and Hofmann, 2011; Young et al., 2019) suggesting similar brain mechanisms in birds and mammals, our knowledge on parental control in birds is limited. The preoptic area in various bird species has been shown to be involved in male sexual behaviours (Merullo et al., 2018; de Bournonville et al., 2019), social stimulus recognition (Lorenzi et al., 2017) and parental care (Slawski and Buntin, 1995; Ruscio and Adkins-Regan, 2004). The expression of amylin, a peptide showing 80% identity to the human amylin (Fan et al., 1994), has been reported in the brain of chicken using RT-PCR (Remes et al., 2015) and Northern blotting (Fan et al., 1994). Amylin receptors have been reported in bird brain, specifically in chicken and the Japanese quail (*Coturnix japonica*), where they were suggested to be involved in food intake and related behaviours (Cline et al., 2010; Yuan et al., 2017). Zebra finch may also contain amylin receptor based on its presence in its genome. Still, the distribution and function of amylin in birds remains to be explored especially in non-precocial birds such as the altricial zebra finch (*Taeniopygia guttata*). Therefore, in the present study, we addressed the distribution of amylin in a biparental passerine bird, the zebra finch. We chose this species for practical reasons; the species breeds well in captivity, and the reproductive behaviours and sex roles are well documented and can be easily established and monitored (Morvai et al., 2016). To obtain information regarding the possible reproductive function of amylin in birds, brains of male and female zebra finches from three different reproductive stages (i.e. when paired without young, incubating eggs or provisioning nestlings) were compared to control birds living in same sex flocks (i.e. non-reproducing unpaired individuals).

## Materials and Methods

### Animals and Keeping Conditions

Subjects were randomly selected from a zebra finch population kept at the animal house of Eötvös Loránd University, Hungary. This captive zebra finch population was established from the domesticated stock maintained at Bielefeld University, Germany (Forstmeier et al., 2007). Birds were ringed by a numbered aluminium ring (Principle Kft., Újlengyel, Hungary). A constant light cycle (lights on from 6:00 AM to 8:00 PM) was kept using full-spectrum light tubes connected to timers. The temperature of the experimental room and humidity level were maintained between 20–21°C, and 55–60%, respectively. The finches were housed in aviaries in same-sex flocks (60–80 birds), so that visual and auditory contact between different-sex flocks was allowed. Food (a seed mixture, supplemental egg-food and germinated seeds) and water were provided *ad libitum*.

The study was carried out according to the Hungarian Laws for the experimentation with animals. Breeding and experimentation was implemented with the permission of the Ethical Board of Eötvös Loránd University (ELTE MÁB 02/2014). A total of 34 birds were used in the study: two were used for RT- PCR and probe preparation, and 32 were used to establish the distribution of amylin at different reproductive stages in both sexes.

### Experimental Groups and Brain Tissue Collection

Brains of 16 adult females and 16 males were examined with *in situ* hybridisation histochemistry in the experiments. Five males and five females stayed in the flocks until tissue sampling (‘non-reproducing’ control group), while 11 females and 11 males were paired randomly. Couples were housed in separate cages (100 × 30 × 35 cm). Wooden nest boxes (12 × 12 × 12 cm) were attached to the cages from outside and coconut fibres were provided as nest material for seven of the 11 pairs. Two of the seven breeding pairs were sacrificed on the 12th day of incubation (‘incubating’ experimental group) whereas the other five pairs were dissected on day 12, post-hatching (‘feeding’ experimental group). The remaining four pairs belonged to the ‘paired’ control group. They were housed as a pair with a nest box attached to their cage but did not receive nesting material. None of these pairs produced clutches or laid any eggs. Birds in this “paired” group were sacrificed in parallel with the incubating parents. For technical reasons, one of the paired control females was not included in the *in situ* hybridisation, resulting in a total of 16 males and 15 females used in the analysis.

The animals were sacrificed by decapitation during daytime, 4–8 h after the onset of the light period. Subsequently, the brains were dissected from the skull and snap frozen in liquid isopentane kept in dry ice. The dissected brains were stored in a −80°C freezer until further processing.

### Preparation of Probes for *in situ* Hybridisation

Preparation of the *in situ* hybridisation probes was performed as described previously (Faber et al., 2007). Hypothalami were dissected from two fresh brains. RNA was isolated using Trizol Reagent (Invitrogen, USA Thermo Fisher Scientific, United States) according to the manufacturer’s instructions. After diluting RNA to 2 μg/μl, it was treated with Amplification Grade DNase I (Invitrogen), and cDNA was synthesised with a Superscript II reverse transcriptase kit (Invitrogen) as described in the kit protocol. After 10-fold dilution, 2.5 μl of the resulting cDNA was used as a template in PCR reactions performed with iTaq DNA polymerase (Bio-Rad Laboratories, Hercules, CA, United States) in total volumes of 12.5 μl under the following conditions: 95°C for 3 min, followed by 35 cycles of 95°C for 0.5 min, 60°C for 0.5 min, and 72°C for 1 min. Primers were used at 300 nM final concentration. The applied primers to isolate two separate parts of zebra finch amylin mRNA (GenBank accession No. XM_002197662.3) were the following: primer pair A: CACCAGCTGGAGAAAAGGAA and ATGCACAGTGGAATGGTGAA, primer pair B: GGATGC TATTGCAGCACCTT and TGCAATGAAGAAAACGGACA. The calculated lengths of the PCR products are 341 and 250 base pairs (bp) for probe A and B, respectively (441–781 and 11–260 bp of GenBank accession No. XM_002197662.3, respectively). The probes share significant sequence homology to the corresponding chicken *Gallus gallus* (GenBank accession No. NM_205397.1: 73% for probe A and 80% for probe B) and mouse *Mus Musculus* (GenBank accession No. NM_010491.2: 62% for probe A and 55% for probe B) amylin mRNA sequences based on BLAST search (NCBI). A comparison of the IAPP (islet amyloid polypeptide) gene in finch (NCBI Gene ID: 100230121) to chicken (Gene ID: 396362), mouse (Gene ID: 15874) and human (Gene ID: 3375) shows high similarities across species: the locus is flanked by SLCO1A2 on one side and PYROXD1 and RECQL on the other, although a bit more distantly in finches. Even though preliminary, this reinforces the hypothesis that the correct orthologue of IAPP has been identified in finches. We also checked potential similar sequences in the zebra finch genome but did not find any other gene, which showed significant similarities to both probes A and B. The primers were chosen to generate probes, which do not overlap so that the comparison of their distributions can be used to assess the specificity of labelling. PCR products were run on a gel, and images were captured with a digital camera. Then, the PCR products were purified from the gel, inserted into TOPO TA cloning vectors (Invitrogen), and transformed chemically into competent bacteria according to the manufacturer’s instructions. Plasmids were purified from five to seven colonies and applied as templates in PCRs with specific primer pairs to select plasmids containing specific inserts. A positive plasmid for each probe was applied as template in PCRs, using primer pairs specific for the probe and also containing T7 RNA polymerase recognition site added to the reverse primers (antisense probes) and T3 RNA recognition site added to the forward primers (sense probes). At the end, the identities of the cDNA probes were verified by sequencing.

### *In situ* Hybridisation Histochemistry

Serial coronal sections (12 μm thick) were cut from 31 whole zebra finch brains using a cryostat, mounted on positively charged slides (SuperfrostPlus^®^, Fisher Scientific, Pittsburgh, PA, United States), dried, and stored at −80°C until use. The brain sections were collected in such a way that consecutive sections were mounted on 18 parallel slides. For *in situ* hybridisation, [^35^S]UTP-labelled riboprobes were generated from the DNA probes using a MAXIscript transcription kit (Ambion, Austin, TX, United States).

The preparation of tissue was performed using an mRNAlocator Kit (Ambion), according to the manufacturer’s instructions. Tissue was prepared using an mRNA-locator Kit (Ambion) according to manufacturer’s instructions. For hybridisation, we used 80 μl hybridisation buffer and 1 million DPM of labelled probe per slide. Washing procedures included a 30 min incubation in RNase A, followed by decreasing concentrations of sodium-citrate buffer (pH = 7.4) at room temperature, and then at 65°C. After drying, slides were dipped in NTB nuclear track emulsion (Eastman Kodak, Rochester, NY, United States), stored for 3 weeks at 4°C for autoradiography, developed with Kodak Dektol developer, fixed with Kodak fixer, counterstained with Giemsa, and coverslipped with Cytoseal 60 (Stephens Scientific, Riverdale, NJ, United States).

### Quantification of the *in situ* Hybridisation Signal

The location of the brain regions was determined based on the stereotaxic atlas for the zebra finch brain (Nixdorf-Bergweiler and Bischof, 2007) as well as on a more detailed atlas for the canary brain (Stokes et al., 1974), with an updated anatomical nomenclature (Reiner et al., 2004). Cells were considered positive if the number of autoradiography dots in them was at least three times higher than the adjacent background. Brain regions with consistent labelling patterns over individuals were identified, and the sections at the same rostrocaudal coordinate were chosen from each individual for the analysis. An Olympus BX51 microscope equipped with a dark-field condensor using a 10x magnification objective was used to find the relevant brain regions and take the photomicrographs for the quantitative analysis. At the ROIs, 5 megapixel, 8 bit greyscale photomicrographs were taken bilaterally. The ROI was always within the borders of the nuclei for the quantitative analysis. In case of sex-dependent size differences, only the area within the nucleus in the particular sex was included in the analysis. The TIFF images were processed and measured using the ImageJ software. First, the background noise was subtracted from all images using background subtract function with a 50 pixel rolling ball radius to eliminate background whose variation could have potentially influenced the grain density measured later. Same size ROIs were defined for every brain region and positioned on every image by hand. Densifications of the *in situ* label (signifying amylin containing cells) were counted within the ROI. To make the measurement comparable in the case of case of sexually dimorphic regions, the ROI was defined to fit within the borders of the region both in males and females. In case of the HVC, we also counted all cells observable within the region both in males and females to test whether the label reflects the size dimorphism of this nucleus. If the density of the label within the area was too high to identify distinct cells and was not possible to count individual grains, we calculated the grain density in a 10 μm diameter circle over each cell by thresholding 8 bit greyscale images automatically using the ImageJ software Renyi entropy function. As a representative of grain density, the fractional area covered by grains was calculated. Fractional area is an accurate estimate of grain number when grain density is high within the ROI (Mize, 1994). The same parameter of a label free region from the same image was also measured on every picture, and was subtracted from the mean grey value of the ROI to control for the background noise over the brain tissue. Label free regions were assigned where no cellular accumulation of the *in situ* label was detected.

In the Ac, Ar.X/lateral rostral St (Ar.X/LrSt) and MLd, grain density over 10 randomly selected amylin positive cells per individual was also measured and averaged to test whether any difference in optical density was a consequence of increased number of cells or more autoradiography label over individual cells. Brightfield photomicrographs were taken using a high magnification objective (40x). Since the label was often extremely dense, it was not possible to count individual grains, so we calculated the optical density in a 10 μm diameter circle over each cell.

### Statistical Analysis

Data of corresponding ROIs were averaged over the two hemispheres and were analysed by a generalised linear model (GLM) using negative binomial distribution for discrete data and gamma distribution for continuous data. Cage number for the pairs and batches *in situ* hybridisation histochemistry experiments were added to the model as random factors. The independent factors were the gender (male, female), and the reproductive status of the individuals. Data from feeding and incubating individuals were pooled since there were only four individuals (two pairs) in the latter group. Therefore, reproductive status consisted of individuals kept in same sex flocks (1), individuals paired without nest material showing no parental behaviour (2) and individuals expressing parental care (incubating eggs or feeding hatchlings) (3). Thus, the data belonged to three groups: flocked, paired and parental; however, data from the incubating and feeding birds are shown separately on Figure 9. Ar.X was also analysed in a separate, univariate model that excluded sex as an independent variable since females lack such a region. Otherwise, the model consisted of the same main effects and interactions as mentioned above. Contrast matrices were used to obtain adjusted *p*-values for the pairwise comparisons among various reproductive stages and gender differences within each reproductive status.

## Results

### Presence of Amylin in the Zebra Finch Brain

**FIGURE 1:**
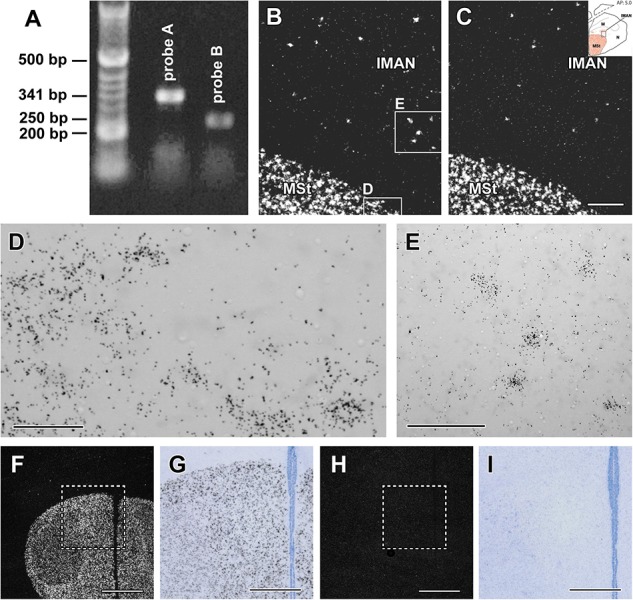
Validation of the *in situ* hybridisation histochemistry procedure. **(A)** Amylin is present in the zebra finch hypothalamus based on reverse transcriptase (RT)-PCR. The non-overlapping DNA sequences within the amylin gene designed to be 341 and 250 bp long, respectively, resulted in the expected length. These sequences were used to design *in situ* hybridisation probes A and B. **(B)** A dark-field photomicrograph of a section containing part of the medial striatum (MSt) and the lateral part of the lateral part of the magnocellular nucleus of the anterior nidopallium (IMAN) demonstrates the clear labelling of amylin mRNA-expressing cells by probe A (white signal) with a low background. **(C)** A dark-field photomicrograph of the same field of an adjacent section labelled with amylin probe B demonstrates the same distribution of amylin mRNA as probe A (B). **(D,E)** High magnification bright-field photomicrographs of the area framed in **B** show the accumulation of individual autoradiography grains (black dots) above cell bodies corresponding to the white signal in **B**. **(F)** A low magnification dark-field photomicrograph of a section of a male zebra finch containing the MSt. Amylin signal is visualised with (antisense) probe A. **(G)** Bright-field photomicrograph of the area framed in **F**. **(H) F:** The same field as in **F** in a section adjacent to the one in **F** hybridised with a radioactive sense probe corresponding to probe A. **(I)** Bright-field photomicrograph of the area framed in **H**. While background labelling is visible, specific labelling is missing completely in **H** and **I**. Scale bars = 200 μm for **C** (and **B**), 50 μm for **D**,100 μm for **E**, 1 mm for **F** and **H** and 500 μm for **G** and **I**.

Both of the two dissected hypothalamic regions contained PCR products of the expected lengths (341 and 250 bp, respectively) for the two primer pairs (Figure 1A). The distribution of amylin mRNA was the same when examined with either of the two probes albeit the longer probe (probe A) yielded somewhat more intense labelling (Figures 1B,C). Therefore, probe A was used to examine amylin at different reproductive periods. Examination of the signal at high magnification revealed the expected Gaussian distribution of the autoradiography grains above the labelled cells (Figures 1D,E).

The specificity of labelling was checked by using sense probes of the same parts of the DNA as for the antisense probes. Specific labelling was absent when the sense probes were used for *in situ* hybridisation histochemistry both for probe A and B. Even when adjacent sections from the same animal were processed together, thus, the only difference being the sequence of the probe (antisense vs. sense), only the antisense probes provided specific labelling (Figures 1F–I). In turn, we detected the same background level for the antisense and sense probes.

### Mapping of Amylin Label

We found topographically organised amylin expression in the brain samples. The overall distribution of the zebra finch amylin mRNA was found to be more widespread and abundant than in the rodent brain (Table 1). Furthermore, unlike in rodents, amylin label was apparent in both sexes, and irrespective of the reproductive status.

**TABLE 1:** Semi-quantitative assessment of amylin levels in brain structures.

**Brain region**	**Abbreviation**	**Label strength**
**Telencephalon**		

**Pallium**		
Apical hyperpallium	HA	+ /++
*Mesopallium*	M	
Dorsal mesopallium	MD	+ +
Ventral mesopallium	MVCe	+
Caudodorsolateral pallium	CDL	+ +
*Nidopallium*	N	
Frontal nidopallium	NF	+
Frontal nidopallium-central region	NFCe	+ +
Basal somatosensory nucleus of the nidopallium	BSS	+ +
Lateral part of the magnocellular nucleus of the anterior nidopallium	lMAN	+ ++/++++
Medial part of the medial anterior neopallium	mMAN	0/ + +
Intermediate nidopallium-superficial region	NIS	+
Caudal nidopallium	NC	+
Caudal nidopallium-medial region	NCM	+ +
HVC	HVC	+ /++
*Amygdala*	Amyg	
Medial amygala		+ ++
Arcopallium		+
Pallial extended amygdala	EA	+ +++
**Subpallium**		
Bed nucleus of the stria terminalis-medial part	BSTm	+ ++
Pallidoseptal transition	PalSe	+ ++++
Striatum	St	
Striatopallidal area	StPal	+ +++
Nucleus accumbens	Ac	+ ++++
Lateral striatum	LSt	+ ++++
Area X (males only)	Ar.X	+
Medial striatum	MSt	+ ++++
Intrapeduncular nucleus	InP	+ ++++
Striopallidal amygdaloid area	StPalA	+ ++
Preoptic area	POA	
Lateral preoptic area	LPO	0/ +
Medial preoptic nucleus	POM	+ +

**Diencephalon**		

**Thalamus**		
Thalamic posteroventral nucleus	PVTh	+
**Prethalamus**		
Reticular nucleus, dorsal part	RtD	+ ++
**Hypothalamus**		
Lateral hypothalamic area	LHy	+ +

**Mesencephalon**		

**Pretectum**		
Medial pretectal nucleus	MPT	+ +
**Mesencephalon**		
Midbrain vocal area (intercollicular area core nucleus)	MVA	+ ++
Interfascicular nucleus	IF	+ +
**Isthmus**		
Isthmooptic nucleus	IsO	+ +

**Rhombencephalon**		

Principal nucleus of the trigeminal nerve	nPrV	+
**Brain region**	**Abbreviation**	**Label strength**

Pedunculotegmental nucleus, superficial part (r1)	PTgS	+
Pontine raphe nucleus (r3,r4)	PnR	+ ++
Reticular formation (r1–r11)	Rt	+
**Cerebellum**		
Granule cell layer of the cerebellum	GrCb	+ ++++

**FIGURE 2:**
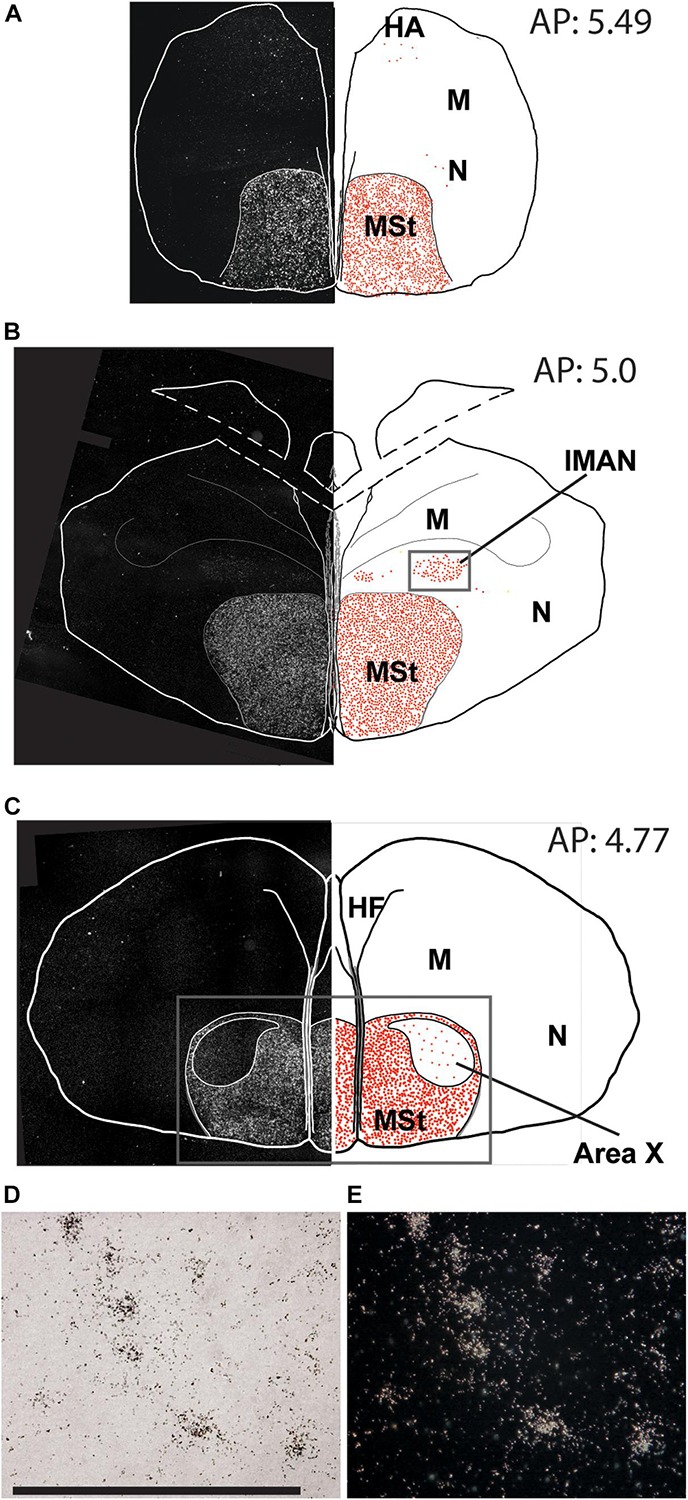
Overview of the topographical distribution of amylin in the rostral regions of the male zebra finch brain. On the left part of the panels, montages of dark-field photomicrographs are shown, which constitute the whole section cut in a frontal plane. The borders of the sections and ventricles are indicated by white lines. On the right part of the panels, drawings of the same brain sections as on the left are illustrated. The names and positions of major brain structures are also indicated. The position of labelled cells are marked by red dots to demonstrate the distribution of amylin mRNA in the sections. The panels **(A–C)** are at different antero-posterior (AP) coordinates: **A** at 5.49 mm, **B** at 5.00 mm and **C** at 4.77 mm. **(D)** A bright-field photomicrograph of the BST shows *in situ* hybridisation labelling in high magnification (scale bar: 100 μm). **(E)** The same field of view as in **D** in dark-field.

**FIGURE 3:**
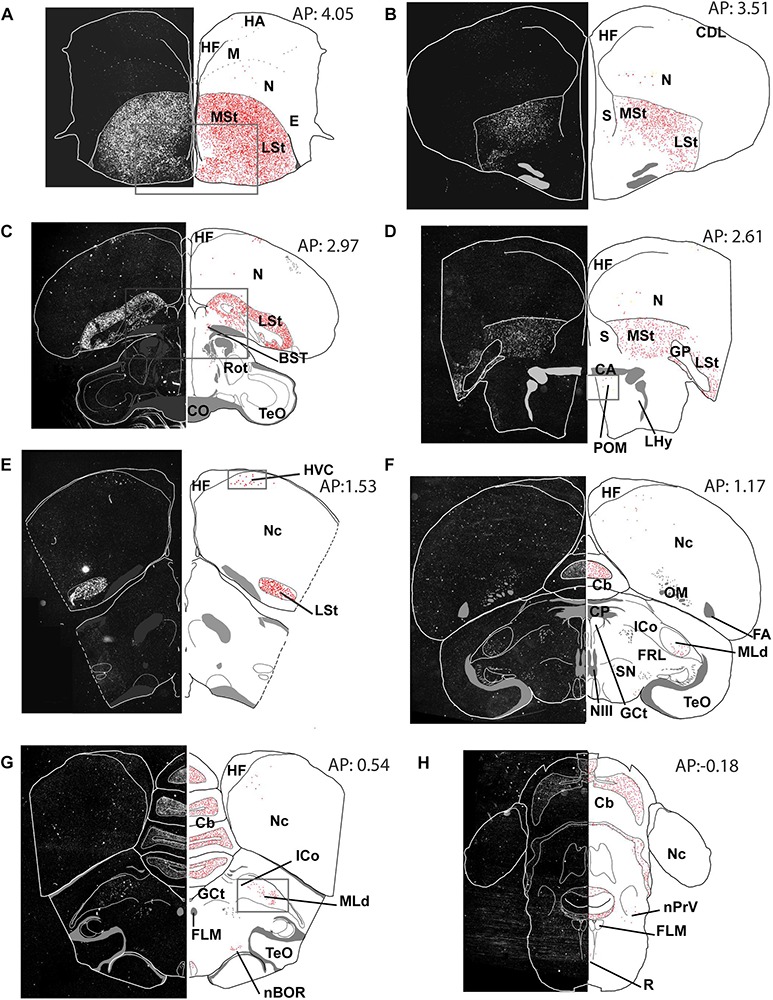
Overview of the topographical distribution of amylin in middle and caudal regions of the male zebra finch brain. On the left part of the panels, montages of dark-field photomicrographs are shown, which constitute the whole section cut in a frontal plane. The borders of the sections and ventricles are indicated by white lines. On the right part of the panels, drawings of the same brain sections as on the left are illustrated. The names and positions of major brain structures are also provided. The positions of labelled cells are indicated as red dots to demonstrate the distribution of amylin mRNA in the sections. The panels **(A–H)** are at different antero-posterior (AP) coordinates: **A** at 4.05 mm, **B** at 3.51 mm, **C** at 2.97 mm, **D** at 2.61 mm, **E** at 1.53 mm, **F** at 1.17 mm, **G** at 0.51 mm and **H** at 0.18 mm.

**FIGURE 4:**
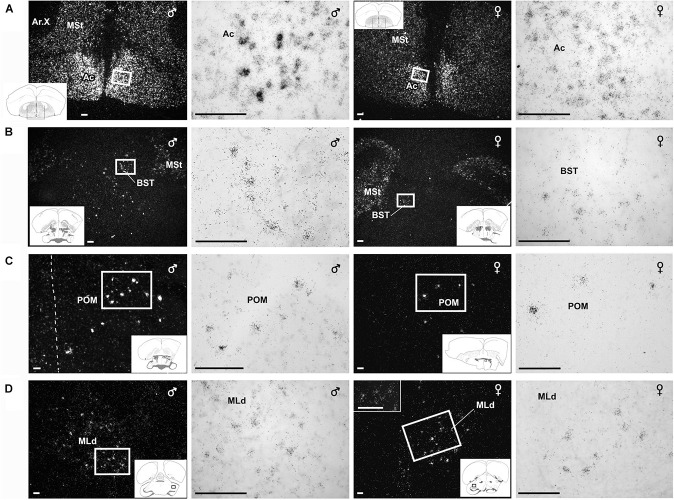
Amylin mRNA in some of the nuclei that belong to the social behavioural network. Photomicrographs show *in situ* hybridisation signal of amylin mRNA taken of frontal sections from brains of feeding individuals in dark- and bright-field. Sections of male brains are shown in the left panels whereas sections from female brains are shown in the right panels. The position of the pictures in low magnification drawings are in the left corner as insets. **(A)** Sections demonstrate particularly high density of amylin-expressing neurons in the accumbens nucleus (Ac) while amylin mRNA is also abundant in the adjacent medial striatum (MSt). The female brain contains considerably less amylin mRNA in the Ac, while the intensity of labelling is similar in the MSt. **(B)** A medium density of intensely labelled amylin-expressing neurons is visible in the bed nucleus of the stria terminalis (BST) of the male zebra finch while amylin mRNA is much less abundant in the female BST. **(C)** A considerable number of neurons expressing amylin mRNA are present in the medial preoptic nucleus (POM). The distributions are similar in males and females; however, the intensity of labelling seems higher in males. **(D)** Amylin-expressing neurons are located in the dorsal part of the lateral mesencephalic nucleus (MLd). The labelling appears similar in males and females. Scale bars = 100 μm.

**FIGURE 5:**
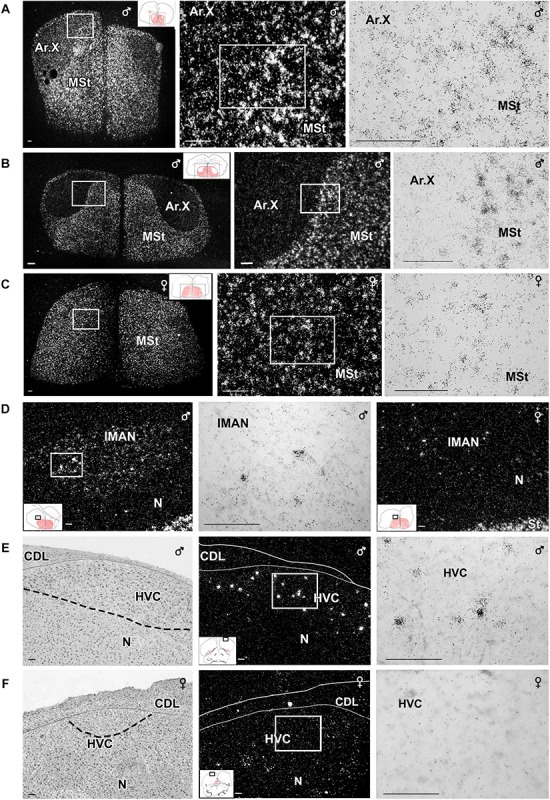
Photomicrographs taken from frontal sections showing *in situ* hybridisation signal of amylin RNA in nuclei belonging to the song system of male and female zebra finch. **(A–C,E,F)** Micrographs in the middle and right panels show enlargements of the areas framed in the panel to their left in dark- and bright-field, respectively. The position of the pictures in low magnification drawings are in the left corner as insets. Medial striatum (MSt) at the level of area X of a paired male **(A)**, a unpaired male **(B)** and the corresponding region of a feeding female **(C)**. The intensity of labelling looks higher in the paired than in the unpaired male Area X, while this nucleus is virtually absent in the female brain. **(D)** Amylin mRNA-expressing neurons in the lateral part of the magnocellular nucleus of the anterior nidopallium (lMAN). A difference in amylin-labelling intensity is visible as the signal looks markedly higher in a paired male male (left and middle) than in a feeding female (right). Amylin expression in the HVC (proper name) in paired male **(E)** and feeding female **(F)**. The left panels are Nissl-stained sections. The same fields of adjacent sections are shown in the middle panels while high-magnification bright-field pictures of the indicated parts in the middle panels are shown in the right panels. Scale bars = 100 μm. Additional abbreviation: M – mesopallium.

**FIGURE 6:**
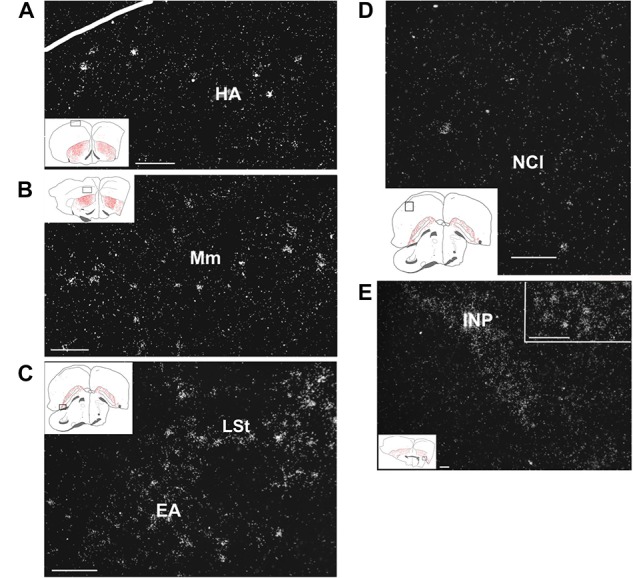
Amylin mRNA in pallial and subpallial telencephalic regions of feeding females. The insets of the left indicate the location of the photomicrographs in the brain. **(A)** Scattered amylin-expressing neurons are visible in the apical hyperpallium (HA). **(B)** The medial mesopallium (Mm) also contains a relatively low density of amylin mRNA-containing neurons. **(C)** A number of amylin-expressing neurons are present in the extended amygdala (EA) and also in the adjacent lateral striatum (LSt). **(D)** A few amylin-positive neurons are scattered in the lateral part of the caudal nidopallium (NCL). **(E)** The intrapeduncular nucleus (INP) contains a high density of neurons with relatively low intensity labelling. The inset in the upper right corner demonstrates that individual neurons can be identified in this brain region as well. Scale bars = 100 μm.

In the telencephalon, amylin mRNA signal was mostly found in the subpallium, particularly in its striatal subdivision (Figures 2, 3A–E). The greatest density was observed in the medial (Figure 4A) and lateral St (Figure 5A) and the interpeduncular nucleus (Figure 6E). In contrast, the GP showed no detectable label (GP; Figure 3D). Strong label was also found in the Ac (Figure 4A). In males, Ar.X of the St was delineated by a weaker level of labelling than the surrounding St (Figure 5A). Nevertheless, the amylin label was still stronger in Ar.X than the general background over brain tissue (Figures 5A,B). The LrSt of the females showed similarly dense amylin label as the medial part (Figure 5C), with no visible anatomical traces of a histologically distinct region such as the putative female Ar.X (Shaughnessy et al., 2019). Amylin mRNA expression was also intense in the caudal lateral part of the BST (Figure 4B), the pallidoseptal transition and the BSTm near the CA (Figure 3C). In the pallium, some amylin mRNA signal was observed in the pallial EA (Figure 6C). This area was named medial amygdala identified by inNkx2.1, Lhx6 and Shh gene expression as well as somatostatin cells (Vicario et al., 2017). It may be the same region, which was also called nucleus Taenia (Reiner et al., 2004). More intense label was apparent in some nuclei related to the song network especially in males: the lMAN (Figure 5B) and the HVC (Figure 5E) showed elevated amylin mRNA level compared to females (Figures 5D,F). Note that the intersex difference in HVC and lMAN on Figure 5 is illustrated by showing a paired male and feeding female. The medial anterior nidopallial nucleus (mMAN) also was labelled. In addition, a few labelled cells also appeared in the HA (Figure 6A), the Mm (Figure 6B), the caudodorsolateral N (Figure 6D), the central region of the frontal and intermediate N, as well as the medial part of the Nc (Figure 3), which corresponds to the auditive field. The scattered cells in these brain regions did not group to form distinct cell populations. Likewise, very few labelled cells were found in the A. There was no label in other telencephalic regions including the nucleus robustus arcopallii and the field L; however, some cells in Nc might be assigned to the latter region.

**FIGURE 7:**
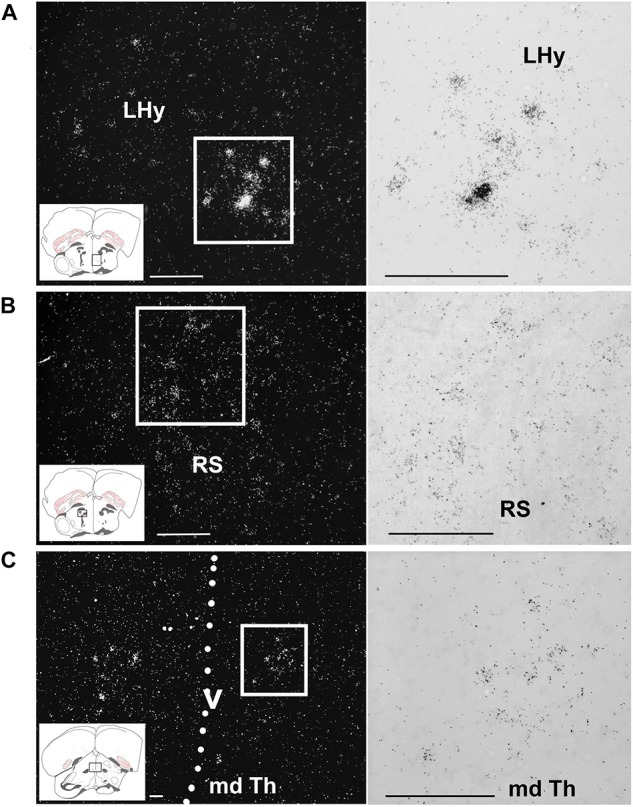
Amylin expression in some diencephalic nuclei of feeding female finches. The insets of the left indicate the location of the photomicrographs in the brain. **(A)** A few intensely labelled amylin-positive neurons are located within the lateral hypothalamus (LHy). **(B)** A medium density but only faintly labelled neurons are present in the superior reticular nucleus (RS) in the thalamus. **(C)** Ventral part of the dorsal medial thalamus (md Th). The dashed line indicates the position of the third ventricle (v). Scale bars = 100 μm.

A moderate signal was found in the POM (Figure 4C) mostly ventral to the CA. In the hypothalamus, the only other notable label was found in a small group of cells in the LHy (Figure 7A). The thalamus appeared devoid of amylin mRNA signal with the exception of the dorsal part of the reticular nucleus (Figure 7B) and a low intensity of labelling in the periventricular and posteroventral nuclei (Figure 7C). The auditory part of the thalamus, such as the nucleus ovoidalis, as well as the amygdalar part of the song system, such as the medial part of the nucleus dorsolateralis anterior and the nucleus dorsomedialis posterior thalami did not exhibit amylin labelling.

**FIGURE 8:**
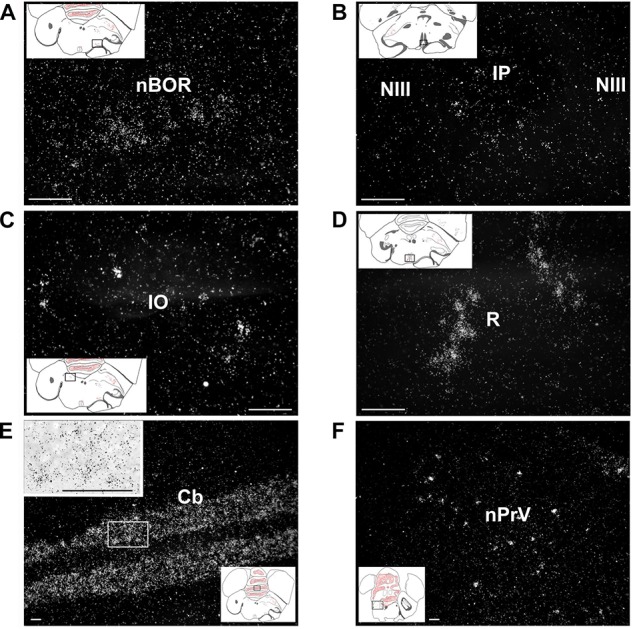
Amylin expression in the brainstem and cerebellum shown in feeding females. The insets indicate the location of the photomicrographs in the brain. **(A)** The nucleus of the basal optic root (NBOR) contains amylin-expressing neurons with relatively low intensity labelling. **(B)** A few amylin-expressing neurons are also found in the rostral interpeduncular nucleus (IP). **(C)** The isthmo-optic nucleus (IO) contains a few amylin-positive neurons with relatively high intensity labelling. **(D)** The raphe nuclei (R) contain a substantial amount of amylin mRNA. **(E)** The granule cell layer of the cerebellum contains a high number of amylin-expressing neurons. The inset in the upper left corner shows the labelling of individual neurons at higher magnification. **(F)** The principal sensory nucleus of the trigeminal nerve (nPRV) contains a medium density of amylin-expressing neurons. Scale bars = 100 μm. Additional abbreviation: NIII – oculomotor nerve.

In the mesencephalon, the amylin RNA signal was concentrated in the midbrain auditory relay nucleus (or midbrain vocal area, MLD; Figure 4D) and the nBOR (Figure 8A). To a smaller extent, amylin mRNA also appeared in the medial pretectal nucleus, the IP (Figure 8B), the interfascicular nucleus and the IO (Figure 8C).

In the rhombencephalon, a moderate signal was present in the pontine R (Figure 8D), whereas some weak labelling was also detected in the parabrachial complex, the nPRV (Figure 8F), the pedunculotegmental area and the reticular formation.

The granule cell layer of the Cb was intensely labelled with the amylin RNA (Figure 8E) while other parts of the Cb did not contain detectable signal.

### Quantitative Analysis

**FIGURE 9:**
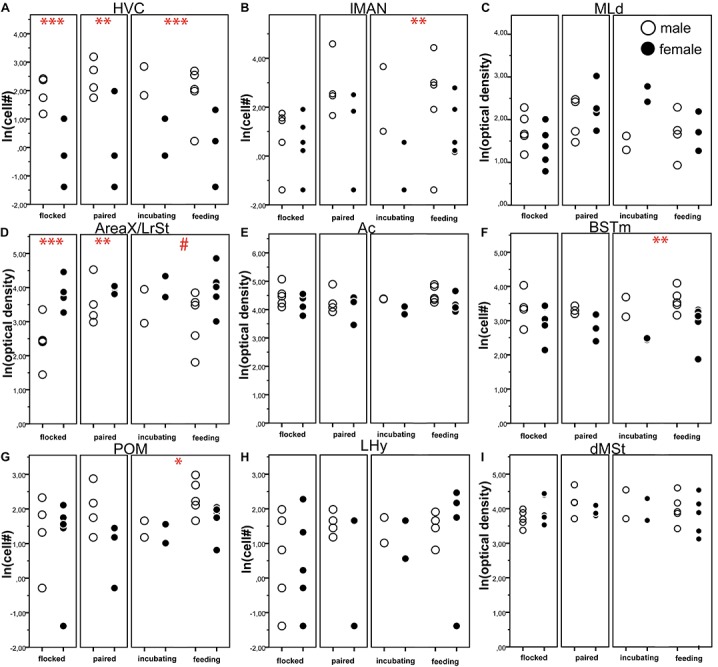
The number of amylin positive cells **(A,B,F–H)** or optical density **(C–E,I)** of amylin label (both ln transformed) in different brain regions of male (open circles) and female (filled circles) zebra finches during various reproductive stages. The circles represent the data of individual birds. Asterisks represent significant difference between the males and females in a specific reproductive stage (^∗^*p* < 0.05, ^∗∗^*p* < 0.01, ^∗∗∗^*p* < 0.001). # represents a trend (0.1 > *p* > 0.05). Data of incubating and feeding birds were merged in the statistical analysis; however, they are presented here separately. Overall, significant sex differences were present in six brain areas (HVC, lMAN, Area X, AC, BSTm, POM) as detailed in Table 2. We also note that Area X in males was not compared to a female Area X, which may not exist, but rather to the lateral rostral striatum of the females.

Some centres of the song production network and the social (decision making) network showed strong sex differences in amylin expression levels. Overall the gender had a significant effect in six out of the nine quantified brain regions (Table 2); however, there were no differences between the social stages in any of the brain regions in either males or females (*z* < 2.04, *p* > 0.220). In the song network, sex had a significant effect on the intensity of amylin label often reflecting the well described size differences among these regions in males and females. Amylin label was stronger in males within the HVC (Figures 5E,F, 9A) and lMAN (Figures 5D, 9B), but not in the MLd (Figures 4D, 9C). Ar.X in males exhibited less intense label when compared to a corresponding region of the LrSt of the females (Figures 5A, 9D) or the neighbouring dmSt of the same brain (*t* = 8.51, *p* < 0.001). No such difference between medial or LrSt was observed in females (*t* = 0.23, *p* = 0.821). In the social brain network, we found strong sex differences (Table 2) in the Ac (Figures 4A, 9E), BSTm (Figures 4B, 9F) and the POM (Figures 4C, 9G). In all of the three regions, amylin label appeared stronger in males. The amylin-positive cell group in the LHy showed no sex difference (Figures 8A, 9H). A region not directly related to social or vocal behaviours, the rostral dmSt (MSt) was also examined but did not show any difference between the sexes (Figures 4A, 9I).

**TABLE 2:** Statistics of sex differences in amylin expression level.

	**Brain area**	**Male vs. female**
Song system	**HVC (cell number)**	***z*_(1,27)_ = 7.59, *p* < 0.001**
	**HVC (cell density)**	***z*_(1,27)_ = 5.31, *p* < 0.001**
	**lMAN**	***z*_(1,27)_ = 3.16, *p* = 0.002**
	MLd	*t*_(1,27)_ = 1.74, *p* = 0.283
	**Ar.X/LrSt^∗^**	***t*_(1,27)_ = 6.13, *p* < 0.001**
Social system	**Ac**	***t*_(1,27)_ = 3.28, *p* = 0.001**
	**BSTm**	***z*_(1,27)_ = 3.63, *p* < 0.001**
	**POM**	***z*_(1,27)_ = 3.43, *p* < 0.001**
	LHy	*z*_(1,27)_ = 0.02, *p* = 0.988
Other	MSt	*t*_(1,27)_ = 0.09, *p* = 0.925

*Significant effects are written in **bold** typeset. ^∗^Area X of males was compared to the corresponding region of the lateral rostral striatum (LrSt) in females.*

Reproductive status had no significant effect on the amylin label in any of the analysed brain regions (Table 1 and Figures 9A–I) probably due to the small sample sizes. However, the sex differences were not equally strong across reproductive stage and brain region: pairwise comparisons in the lMAN, BSTm and POM revealed that in these regions sex differences were detectable only in the parental phase (Figures 9B,F,G) On the other hand, HVC showed massive sexual dimorphism in the amylin label irrespective to the reproductive stage either when all labelled cells were counted at its largest extent (Figure 9A) or when cell density was measured in a smaller subset of the nucleus that fit within the borders of the region both in males and females (Table 2) The low amylin expression of the Ar.X in males, however, differed from the corresponding female LrSt significantly only in the flocked (unpaired) and paired finches. The difference was reduced in the parental group and became a non-significant trend (Figure 9D). Grain density over the cells of Ar.X in males (11.72 ± 1.31) was significantly lower (*t* = 8.38, *p* < 0.001) than in the LrSt of females (23.00 ± 2.18). In contrast, in the Ac, the individual cells were equally strongly labelled in both sexes (males: 39.79 ± 1.66, females: 36.41 ± 2.35, *t* = 1.26, *p* = 0.206) suggesting that the overall density difference in the Ac is due to a higher density of cells rather than a higher expression of amylin in individual cells. In the MLd, there was also no difference in the grain density over individual cells.

## Discussion

The results first identified amylin (or islet amyloid polypeptide) as a gene expressed in the zebra finch brain. The distribution of amylin has also been determined in the present study. We discuss the consequences of its more widespread expression pattern than previously described in the rodent brain. In addition, functional implications of the sexually dimorphic expression pattern of amylin are also presented.

### The Presence of Amylin in the Zebra Finch Brain

The high degree of amylin sequence identity between rodent and bird species (over 55% in the region of the probes) suggests that we have correctly identified the zebra finch amylin orthologue. The expression of amylin in the zebra finch brain was first established by RT-PCR in the study. These results are in line with a previous report that amylin mRNA is present in the chicken brain (Remes et al., 2015) and suggest that amylin may be expressed in the brain of different bird species. Furthermore, we provided a more precise determination of the location of the expression of amylin by *in situ* hybridisation histochemistry. The strong accumulation of autoradiography grains above some but not all cells suggest that the labelled cells contain a considerable amount of amylin mRNA while most brain cells are devoid of labelling above background level suggesting that they do not express amylin. The lack of zebra finch sequences homologue to the two (antisense) amylin probes and the same brain distribution pattern using the two non-overlapping probes as well as the absence of labelling for sense probes ensure specific labelling in our experiments. The distribution of amylin was topographically organised as it was clustered in specific brain regions and not present in others. Furthermore, some brain regions containing amylin showed high density of labelled cells (e.g. St) while amylin was present only in some scattered cells in other brain regions and not expressed at all in most parts of the brain. Such a topographic distribution suggests specific functions of amylin in concerned brain areas. Since amylin in rodents is a secretory peptide, and zebra finch amylin has significant sequence homology to both the mouse and chicken amylin gene, we can assume that it is a neuropeptide in the zebra finch brain as well, and possesses neuromodulatory functions. In fact, amylin mRNA has been reported in the brain of the chicken by Northern blotting (Fan et al., 1994). Amylin may exert its potential modulatory actions via the amylin receptor, which has been detected in the brain of chicken and the Japanese quail (*C. japonica*) implying that the receptor may also be present in the zebra finch based on its presence in the zebra finch genome. No detailed mapping of the amylin or its receptor is available in precocial birds, therefore the amylin expression found in the zebra finch might be specific to altricial birds rather than a general pattern in the avian species. Thus, amylin, which was first demonstrated to be a neuropeptide in the rat only relatively recently (Dobolyi, 2009), may be a new member of the zebra finch neuropeptidome (Xie et al., 2010).

### Potential Functions of Amylin Based on Its Brain Distribution

In the rat, amylin is expressed only in the preoptic area of mothers suggesting its role in the control of maternal behaviours. Based on our results, amylin expression is much more widespread in the zebra finch brain, which suggests its involvement in more diverse functions and/or it might reflect a more fundamental evolutionary difference between sauropsida and mammals. In the telencephalon, amylin is particularly abundant in striatal area, both the medial and lateral St as well as the accumbens nucleus. Since these structures serve the same functions in birds and mammals (Reiner et al., 2005), these locations suggest that amylin may be involved in the control of movement, learning as well as motivated behaviours. The high density of amylin-expressing cells in these brain regions suggests that amylin is expressed by the abundant projection neuronal cell type, the GABA-ergic medium spiny neurons (Kuenzel et al., 2011; Kardos et al., 2019). However, amylin is not a ubiquitous neuropeptide of all subpallial motor structures as it is absent in the GP suggesting its specific striatal functions. As opposed to the widespread expression of amylin in the St, it has a highly restricted expression in pallial structures. Based on the position of amylin-expressing pallial neurons, its involvement in song learning may be important as discussed below. Furthermore, amylin may also be present in different amygdaloid structures where it could play a role in emotional processing (Cheng et al., 1999; Papini et al., 2018; Fazekas et al., 2019).

The diencephalic expression of amylin was found to be highly restricted to a few brain regions. One of them was the medial POM, which is known to be involved in social and reproductive functions. It is worth mentioning that in rodents, the medial preoptic area including the POM and the adjacent BST was the only site of amylin expression with potential function in parental behaviour (Szabó et al., 2012). Therefore, it is conceivable that zebra finch amylin in the POM and the BST may also be involved in parental behaviour even if amylin expression is not confined temporally to the period of offspring provisioning as in rodents. The fact that in the present study POM and BSTm were two of the three regions where sexual dimorphism in the amylin expression was most articulated during the parental stage of the reproductive cycle (Figure 9) also supports this hypothesis. Alternatively, as the POM, BSTm and Ac are all members of the ‘social brain network’ (Newman, 1999), or in a wider sense the ‘social decision making network’ (O’Connell and Hofmann, 2011, 2012), amylin could be involved in more diverse social behaviours than parenting in rodents. In turn, the expression of amylin was somewhat higher in these structures in males than in females suggesting a sexually dimorphic action of amylin on the social behaviours.

Additional expression of amylin in the LHy, different thalamic, brainstem and cerebellar structures suggests that the neuropeptide could be involved in a variety of additional brain functions. Based on the relatively restricted expression pattern of amylin, its specific rather than general neuronal functions are expected. One potential role of amylin could be the control of food intake and energy metabolism since some functional evidence of such function is available in mammals (Mietlicki-Baase and Hayes, 2014) and birds (Yuan et al., 2017). The precise brain regions involved are not yet known; however, the anorexic effect of amylin seems to be evolutionarily conserved at least in the amniote lineage. Central administration of amylin has anorexigenic effect in rats (Rushing et al., 2002; Mollet et al., 2004), domestic chicks (Cline et al., 2008) and quails (Yuan et al., 2017). These functions may be exerted by the currently unknown projections of the amylin-expressing neurons to different feeding centres of the brain.

### Sexually Dimorphic Expression of Amylin in the Song System Suggests Its Modulatory Role in Song Learning

Several amylin-expressing nuclei (HVC, lMAN, Ar.X, MLd) are part of the so called ‘song system’ (Mooney, 2009), which is responsible for the detection, learning and production of vocal signals in oscine birds. In fact, the amylin-expressing nuclei all belong to the so-called anterior forebrain song system responsible for the learning of song rather than just motor execution (Mooney, 2009). Since song learning occurs only in male zebra finches, such a widespread, sexually dimorphic expression pattern of amylin in various nuclei belonging to the anterior forebrain song system suggests that amylin can modulate vocal motor learning or the generation of learned songs. Many of the regions participating in vocal communication show marked sex differences: a higher level of expression in males. The only exception is Ar.X, a region earlier thought to be male specific (Nottebohm and Arnold, 1976; Nixdorf-Bergweiler, 1996), however, recently found in females as well although in a much less developed form (Gahr, 2007; Shaughnessy et al., 2019). Ar.X also expresses amylin in males although the label is weaker than those of the neighbouring striatal regions or the corresponding LrSt of the females. The sex difference here is not the lack of amylin-positive cells in males but the much lower expression of amylin mRNA in individual Ar.X cells. Moreover, in Ar.X/LrSt, the difference between females and males is reduced compared to the massive intersex difference in the flocked (unpaired) or paired birds. Such a biased amylin expression in the vocal system of males is in line with the well-known characteristic of zebra finch whose singing is present only in males (Arnold et al., 1986; Elie and Theunissen, 2016) and suggests that amylin may influence song learning and/or promote changes in male singing. Thus, amylin is a novel member of neuropeptides with sex-specific expression level in the song system, which may contribute to the fine-tuning of the complex singing behaviour (Bottjer et al., 1997; Lovell et al., 2008). As to the nature of this change, it is noteworthy that in Ar.X, and to a lesser degree lMAN, amylin mRNA levels were especially higher in incubating and feeding males as opposed to females but no such difference was evident in the flocked (unpaired) birds. It is well established that vocalisation changes after pair formation (D’Amelio et al., 2017). Acoustic communication ceases to function as a sexual display and becomes an agent for coordination of behaviour required for biparental care (Boucaud et al., 2017). Parallel to these changes in the vocalisation, the ratio of female-directed and undirected (advertising) songs (Zann, 1996) is also altered during the reproductive cycle and female-directed singing activates immediate early gene expression in the Ar.X and lMAN much less than undirected singing (Jarvis et al., 1998). Since female directed singing is expected to be more frequent in paired, incubating and feeding males, the higher amylin expression in the Ar.X and lMAN during these stages but not in flocked males might be related to the activation of these areas during female directed singing. The observed pattern of amylin expression in the male HVC suggests that only a small subpopulation of the HVC neurones express amylin, which may or may not overlap with one of the two projection neuron types which send fibres to Ar.X (Bottjer, 2004; Brainard, 2004) and the RA (Nottebohm et al., 1982; Johnson and Bottjer, 1993), or HVC interneurons, and double-labelling experiments to determine which HVC cell types express amylin might reveal a specific function of amylin in song production.

## Conclusion

Considering the widespread but rather specific expression of amylin together with its social environment and sex-dependent changes, this neuropeptide might participate in the organisation of various social behaviours especially via the control of vocal communication. Amylin increases during parental care in males compared to females in some regions of the social network and in some regions of the song network, which is more or less the opposite to what was found in rats (Dobolyi, 2009). Such changes in amylin expression might play a role in the attenuation of the male specific vocal and social behaviours after mating.

## Data Availability Statement

The datasets generated for this study are available on request to the corresponding author.

## Ethics Statement

The animal study was reviewed and approved by the Ethical Board of Eötvös Loránd University.

## Author Contributions

GZ participated in the design of the experiments, data acquisition and data processing, the histological analysis, performed the quantitative analysis, and wrote the first version of the manuscript. CM participated in histological and data analysis, and prepared histological figures. EF participated in the collection of zebra finches and the *in situ* hybridisation histochemistry procedures. RK and SP participated in the statistical analysis of the data. FD participated in the preparation of *in situ* hybridisation probes. ÉR participated in the production of *in situ* hybridisation probes and the *in situ* hybridisation histochemistry procedures. AC participated in the evaluation of the study and contributed to the manuscript. ÁP participated in the design of the experiments, maintenance and collection of the zebra finches, and contributed to the manuscript. AD participated in the design of the study, the probe preparation and *in situ* hybridisation histochemistry procedures, the analysis and interpretation of the data, and the writing of the manuscript.

## Conflict of Interest

The authors declare that the research was conducted in the absence of any commercial or financial relationships that could be construed as a potential conflict of interest.

## Abbreviations

A: arcopallium
Ac: nucleus accumbens
Ar.X: Area X
BST: bed nucleus of the stria terminalis
BSTm: medial part of the bed nucleus of the stria terminalis
CA: anterior commissure
Cb: cerebellum
CDL: dorsolateral corticoid area
CO: optic chiasm
CP: posterior commissure
dmSt: dorsomedial striatum
E: entopallium
EA: extended amygdala
FA: frontoamygdaloid tract
FLM: longitudinal medial tract
FRL: lateral reticular formation
GCt: central grey
GP: globus pallidus
HA: apical hyperpallium
HF: hippocampal formation
HVC: proper name
INP: intrapeduncular nucleus
IO: isthmo-optic nucleus
IP: interpeduncular nucleus
ICo: intercollicular nucleus
LHy: lateral hypothalamic area
lMAN: lateral part of the magnocellular nucleus of the anterior nidopallium
LSt: lateral striatum
LrSt: lateral rostral striatum
M: mesopallium
MAN: medial anterior nidopallium
Md.Th: mediodorsal thalamus
MLd: the dorsal part of the lateral mesencephalic nucleus
Mm: medial mesopallium
MSt: medial striatum
N: nidopallium
nBOR: nucleus of the basal optic root/nucleus ectomamamillaris
Nc: caudal nidopallium
NCL: caudalateral nidopallium
NIII: oculomotor nerve
nPrV: principal nucleus of the trigeminal nerve
OM: tractus occipito-mesencephalicus
POM: medial preoptic nucleus
R: raphe nucleus
ROI: region of interest
Rot: nucleus rotundus
RS: thalamic superior reticular nucleus
RT-PCR: real-time polymerase chain reaction
S: septum
SN: substantia nigra
St: striatum
TeO: optic tectum.
